# A Case of Amelanotic Malignant Melanoma of the Lingual Base That Was Diagnosed Based on a Biopsy of Late Metastasis to a Lumbar Vertebra after Being Misdiagnosed as HPV-Positive Oropharyngeal Anterior Wall Squamous Cell Carcinoma

**DOI:** 10.1155/2021/7139280

**Published:** 2021-09-30

**Authors:** Takumi Okuda, Shinsuke Ide, Kei Kajihara, Tetsuya Tono

**Affiliations:** Department of Otolaryngology-Head and Neck Surger, Faculty of Medicine University of Miyazak, Kihara 5200,Kiyotake, Miyazaki 889-1692, Japan

## Abstract

We report a case of amelanotic malignant melanoma (AMM) in a 66-year-old female. AMM of the lingual base was diagnosed based on a biopsy of late metastasis to the bone marrow of the L4 lumbar vertebra. The patient was initially treated with chemoradiotherapy after being misdiagnosed with poorly differentiated human papillomavirus- (HPV-) related squamous cell carcinoma of the oropharyngeal anterior wall. p16 immunostaining is used to diagnose HPV-related oropharyngeal cancer. However, while p16 expression is used as a surrogate marker of HPV infection, it is important to be aware that p16 protein overexpression can also be caused by other factors. Malignant melanoma is known to express the p16 protein. Morphologically differentiating between AMM and poorly differentiated squamous cell carcinoma based on hematoxylin-eosin staining is difficult. Therefore, in cases that are pathologically diagnosed as p16-positive poorly differentiated oropharyngeal squamous cell carcinoma, it is important to rule out AMM.

## 1. Introduction

In the eighth edition of the TNM classification (AJCC/UICC), which was published in 2017, the section about head and neck cancer was revised markedly. The classification of oropharyngeal cancer into human papillomavirus- (HPV-) related oropharyngeal cancer and non-HPV-related oropharyngeal cancer was particularly important [1, 2]. This change was based on the concept that HPV-related cancer is biologically and prognostically different from conventional oropharyngeal cancer derived from drinking and/or smoking. Due to its versatility and low cost, p16 immunostaining is used to aid the diagnosis of HPV-related oropharyngeal cancer, as p16 expression is considered to be a surrogate marker of HPV infection. However, p16 protein overexpression can be caused by factors other than HPV infections. Malignant melanoma is known to express the p16 protein although the mechanism responsible for this is unclear [3]. On the other hand, it is difficult to morphologically differentiate amelanotic malignant melanoma (AMM) from poorly differentiated squamous cell carcinoma based on hematoxylin-eosin (HE) staining. Therefore, it is important to rule out AMM in cases in which the pathological diagnosis is p16-positive poorly differentiated oropharyngeal squamous cell carcinoma. We report a case of AMM of the lingual base that was diagnosed based on a biopsy of late metastasis to the bone marrow of a lumbar vertebra. The patient was initially treated with chemoradiotherapy after being misdiagnosed with HPV-related poorly differentiated squamous cell carcinoma of the oropharyngeal anterior wall. To the best of our knowledge, this is the first time such a case has been reported. This was an extremely rare case, but it reminded us that, during the diagnosis of oropharyngeal cancer, it is important to bear in mind that p16 expression is not the only surrogate marker of HPV infection.

## 2. Case Report

**Figure 1:**
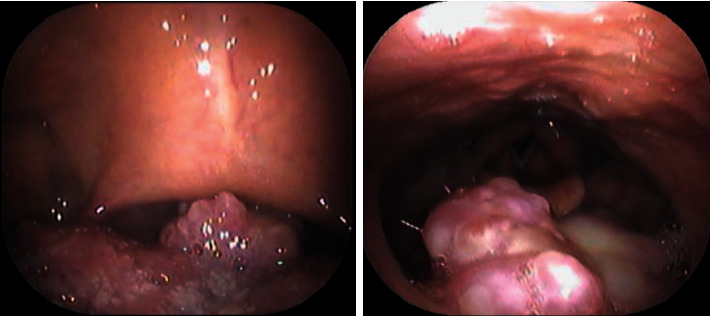
An endoscopic examination of the pharynx showed an irregularly elevated lesion at the base of the tongue.

**Figure 2:**
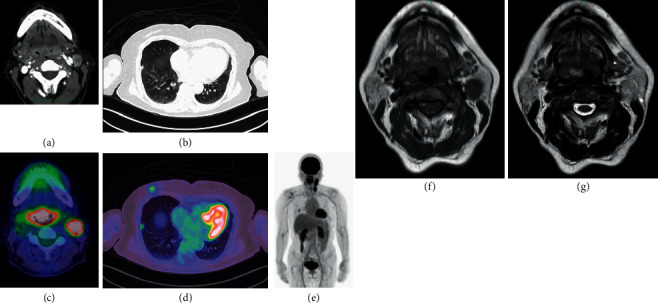
(a) Neck CT showed a tumor at the base of the tongue and left cervical lymph node metastasis. (b) Chest CT showed a nodule in the right inferior lobe of the lung and a postoperative scar lesion derived from breast cancer on the right chest wall. (c) PET-CT showed marked glucose uptake by the base of the tongue (maximum standardized uptake value (SUV max) early: 16.35, delayed: 17.90), the left cervical lymph nodes (SUV max early: 15∼17, delayed: 17∼19), (d) the scar on the right chest wall (SUV max early: 3.47, delayed: 3.19), and the nodule in the right lung (SUV max early: 2.09, delayed: 2.42). (e) There was no other apparent distant metastasis. (f, g) Neck MRI T1-weighted imaging (WI) and T2WI showed that the tumor at the base of the tongue and the left cervical lymph nodes exhibited slightly higher intensity than the surrounding muscles.

**Figure 3:**
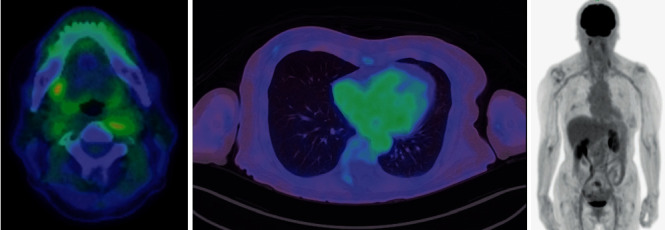
At 6 months after treatment, PET-CT no longer showed increased glucose uptake by the lesions.

**Figure 4:**
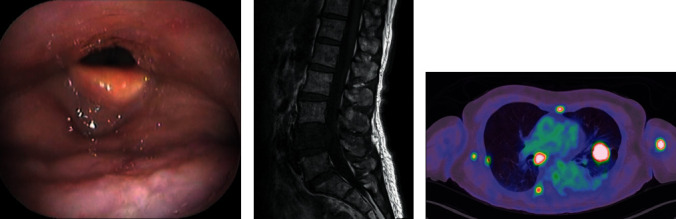
At 20 months after treatment, (a) an endoscopic examination of the pharynx showed that there was no recurrence at the base of the tongue. (b) Lumbar MRI T1W1 showed a metastatic lesion in the L4 vertebra. (c) PET-CT showed multiple metastases in the bilateral lungs, right hilum, right axillary lymph nodes, and some bones.

**Figure 5:**
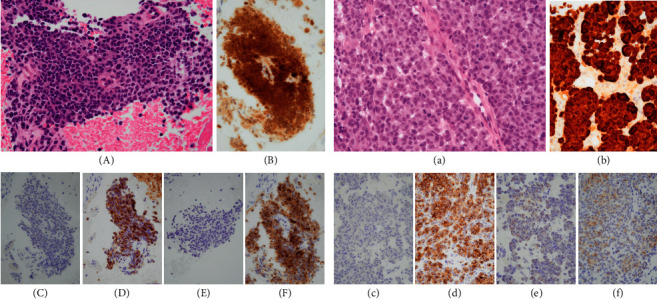
The microscopic findings of the resected L4 bone marrow (A) and pharyngeal tumor (a) are shown. HE staining did not reveal melanotic granules in the malignant cells of either lesion. Proliferating atypical cuboidal cells with hyperchromatic nuclei and nucleoli were distributed in small nests or as single cells and had invaded the stroma. No apparent intercellular bridges or keratinization was observed. Immunohistochemically, the L4 bone marrow tumor cells were diffusely positive for p16 (B). They also produced positive results during HMB-45 (D) and melan-A (F) staining, but negative results during AE1/AE3 (c) and S-100 (e) staining. Similarly, the pharyngeal tumor cells were diffusely positive for p16 (b). They also produced partially positive results during HMB-45 (d), S-100 (e), and melan-A (f) staining and negative results during AE1/AE3 (c) staining.

A 66-year-old female visited our dental hospital because of lymphadenopathy on the left side of her neck and swelling of the left maxillary gingiva one month before our first medical examination. She received a diagnosis of periodontitis and lymphadenitis, and the former was relieved by antimicrobial treatment. However, she was referred to our department to undergo a medical examination because a mass was found at the lingual base, and the left neck lymphadenopathy had not improved. An endoscopic examination of the pharynx showed an irregularly elevated lesion on the base of the tongue (Figure 1). A biopsy of the mass resulted in a pathological diagnosis of p16-positive poorly differentiated squamous cell carcinoma. Right lung metastasis was observed on chest computed tomography (CT) and positron emission tomography- (PET-) CT (Figure 2), and HPV-positive poorly differentiated squamous cell carcinoma of the oropharyngeal anterior wall (T2N1M1 stage IV) was diagnosed. The patient's blood squamous cell carcinoma antigen level before treatment was 0.7 ng/ml. She had undergone surgery for right breast cancer (pT1aN0M0 stage I) one year ago and had received endocrine therapy. There was nothing important in her family history. She had a history of smoking (20 cigarettes/day for 20 years) and drinking alcohol. We treated her with chemoradiotherapy (CRT). Because in this case, as the pathological diagnosis was HPV-positive squamous cell carcinoma, it was thought that the primary and neck lesions were more likely to be controlled by CRT. Besides, the distant metastasis was only to the right lung and it was a single lesion and small of around 1 cm. Therefore, we thought the lung metastatic lesion could be controlled by video-assisted thoracoscopic surgery (VATS) after local treatment. Actually, we had the experience of such a case before. The planned regimen included two cycles of cisplatin (100 mg/m^2^ on days 1 and 23) with 66 Gy of radiation in 33 fractions of 2 Gy per fraction beginning on day 1. Furthermore, in order to reduce the lung metastasis, 3 cycles of enforced FP therapy (cisplatin (100 mg/m^2^ on day 1) and fluorouracil (1000 mg/m^2^ on days 1 through 4)) were administered as additional chemotherapy. PET-CT performed after 6 months' treatment showed that the primary lesion in the oropharynx, the metastasis to the neck, and the pulmonary metastasis had disappeared (Figure 3). However, multiple (bilateral) pulmonary metastases were seen on chest CT conducted at 10 months after treatment. We gave the patient 10 cycles of nivolumab (240 mg/body, every other week), but it aggravated her condition. We administered cetuximab alone (250 mg/m^2^, weekly) for 16 cycles because myelosuppression occurred after 2 cycles of paclitaxel (80 mg/m^2^) and cetuximab (400 mg/m^2^ on day 1; 250 mg/m^2^ on day 8). Metastasis to the fourth lumbar vertebra and worsening of the pulmonary metastasis were seen on lumbar magnetic resonance imaging (MRI) and PET-CT performed 20 months after the chemoradiotherapy (Figure 4). As the metastasis to the lumbar vertebra was causing lower back pain, a biopsy of the lesion was performed by the radiologist at our hospital in conjunction with percutaneous vertebroplasty. Based on the histopathology of HE-stained biopsy specimens, it was considered unlikely that the lesion was an epithelial tumor, and immunostaining was performed. As a result, it was diagnosed as a metastatic lesion derived from malignant melanoma because epithelial marker staining produced negative results, and HMB-45 and melan-A staining were positive (Figure 5). A reexamination of the biopsy specimens of the original oropharyngeal tumor resulted in a final diagnosis of AMM. As we did not detect a primary lesion in the skin during a whole-body search performed by a dermatologist at our hospital, we diagnosed it as AMM of the oropharynx, i.e., the upper aerodigestive tract. Therefore, it was considered that the original disease should have been classified as T4aN2M1 stage IV (according to the AJCC/UICC eighth edition).

## 3. Discussion

The p16 gene, which is also known as the cyclin-dependent kinase inhibitor 2A (CDKN2A) gene, was discovered as a tumor-suppressor gene, located on the short arm of chromosome 9, in 1994 [4]. p16 gene mutations have been found in many malignant tumors and have attracted attention as a cause of familial malignant melanoma [5]. In nonfamilial malignant melanoma, it has been reported that p16 gene mutations contribute to early tumor progression [6]. It is generally considered that p16 gene mutations result in reduced p16 expression in neoplastic cells. However, in cutaneous malignant melanoma, it has been reported that the p16 protein was not detected during immunohistochemistry in about 15% (6/39) of cases, indicating that p16 gene inactivation is not very common [6]. On the other hand, Prasad et al. reported that p16 protein expression was detected in about 25% (19/76) of cases of malignant melanoma of the mucous membranes in the head and neck region [3]. In addition, in a study about oral malignant melanoma, p16 protein expression was detected in about half of cases, but it was not apparent whether it was a prognostic factor [7]. In a domestic study, p16 protein expression was detected in 71% (10/14) of cases of malignant melanoma of the oral mucosa, but it did not have a significant effect on prognosis, or the frequency of local recurrence, cervical lymph node metastasis, or distant metastasis. It was reported that p16 protein expression decreased with tumor progression [8]. The p16 gene mutations associated with malignant melanoma and the factors that affect p16 protein expression remain unknown. In contrast, in HPV-related oropharyngeal cancer, increased p16 protein expression occurs due to infection of the pharyngeal mucosa by HPV. HPV causes infected cells to produce the E7 protein, which inactivates the Rb protein, a product of the tumor-suppressor gene RB1 [9]. The p16 protein downregulates Rb protein expression, and we can detect HPV infections indirectly by detecting the p16 protein, as it is overexpressed in HPV-infected cells [10]. This is the reason why p16 immunostaining is used to facilitate the diagnosis of HPV-related oropharyngeal cancer.

Furthermore, it is difficult to morphologically differentiate poorly differentiated squamous cell carcinoma from AMM based on HE staining [11–13]. Therefore, in cases in which oropharyngeal cancer is pathologically diagnosed as p16-positive poorly differentiated squamous cell carcinoma, it is important to rule out AMM. When HE staining is employed for histopathological examinations, the findings of malignant melanoma resemble those of malignant fibrous histocyte tumors, poorly differentiated cancer, anaplastic carcinoma, and malignant lymphoma. If melanin granules are present, diagnosing malignant melanoma is easy, but cases like the present one are difficult to diagnose. In the present case, we asked a pathologist to perform an examination of the pharyngeal lesion, including p16 staining and an evaluation of malignancy. As a result, the pathologist made an initial diagnosis of p16-positive poorly differentiated squamous cell carcinoma based on histopathological HE staining and p16 immunostaining. If we had mentioned the possibility of AMM as a differential diagnosis, the pathologist may not have made this mistake.

AMM is relatively rare, accounting for about 2–8% of all malignant melanomas [14–16], and the prognosis of AMM is worse than that of the melanotic type of malignant melanoma. The 5-year survival rate of AMM is 36%, while that of the melanotic type is 69% [17]. The reason why AMM has a poor prognosis is unclear, but the following possibilities have been suggested. It may be that the neoplastic cells of malignant melanoma lose their melanin-producing ability. In addition, as AMM is difficult to diagnose, it is often detected late, and misdiagnosis can result in inappropriate treatment being provided. Also, pathologically, many cases of AMM belong to the nodular type, which is highly malignant [14, 17]. The reason why AMM does not produce melanin is still unknown, but there have been various suggestions. One possibility is that AMM exhibits decreased or no synthesis of tyrosinase, which is an essential enzyme in the melanin synthetic pathway. Alternatively, the mechanism responsible for delivering tyrosinase to melanosomes may be defective in AMM. Another possibility is that a tyrosinase inhibitor inactivates tyrosinase [14, 18].

In the current case, we did not detect abnormal radiotracer accumulation in the skin on PET before treatment, and even a whole-body search conducted by a dermatologist at our hospital after the final pathological examination did not reveal any lesions in the skin. Therefore, the final diagnosis was AMM of the oropharynx (i.e., the upper aerodigestive tract). However, the possibility that an unknown primary malignant melanoma dissipated naturally cannot be ruled out. In total, 2.6% of malignant melanoma cases are considered to involve unknown primary lesions [19]. In addition, malignant melanoma can dissipate naturally, and this has been reported to occur in 4–10% of cases [11, 17]. It was reported that PET is useful for staging malignant melanoma and discovering very-early-stage lesions that are undetectable by conventional modalities, such as CT and magnetic resonance imaging (MRI) [20, 21]. Holder et al. reported that the sensitivity and specificity of ^18^F-fluorodeoxyglucose-PET for detecting metastases from malignant melanoma are 94.2% and 83.3%, respectively; therefore, it is a useful modality [20]. However, its sensitivity decreases for lesions measuring ≤6 mm in diameter [21]. In the current case, no skin lesions were detected on PET-CT before treatment. There are two possible explanations for this. The first is that any lesions were extremely small, and the other is that malignant melanoma that arose in the skin disappeared naturally after having metastasized [22–24]. Regarding the metastasis of malignant melanoma, Honda et al. [24] reported that 32 of 46 cases (70%) in which metastasis was discovered involved lymph node metastasis, and the primary lesion was not identified in 35 cases (76%). In the present case, based on the fact that the original lesion was located in the pharynx and metastasis to the regional lymph nodes occurred, it was considered that the oropharynx anterior wall was the primary site.

Although the disease in the present case was AMM, the CRT administered after the patient was misdiagnosed with squamous cell carcinoma produced a complete response (CR) for the head and neck lesions and the distant metastasis to the lungs. Although multiple distant metastases to the lungs, bones, and liver were subsequently detected, the head and neck lesions remained in a state of CR. Conventionally, malignant melanoma is considered to exhibit low radiosensitivity, and many studies have found that X-ray-based radiotherapy is inappropriate. However, there have been various reports about increased doses of radioactivity/daily doses of radioactivity resulting in improved treatment outcomes. Thompson et al. [25] reported that hypofractionated radiotherapy involving a large dose of radioactivity (6–8 Gy) resulted in an efficacy rate of about 80%. In addition, Albertsson et al. [26] reported that combining accelerated hyperfractionated radiotherapy, in which a small dose of radioactivity (1.5 Gy) was administered twice daily, with CDDP chemotherapy resulted in an efficacy rate of about 83% (5 of 6 cases). On the other hand, our method involved normal radiotherapy fractions; i.e., the patient was irradiated with 2 Gy once a day 33 times, combined with CDDP chemotherapy (100 mg/m^2^) repeated every 3 weeks. We can only say that it was very lucky that a CR was achieved, even if it was temporary. As for the reason for the CR, it is considered that CDDP demonstrates relatively strong activity against malignant melanoma although it is not superior to dacarbazine [27]. Another possibility is that the radiotherapy had abscopal effects [28].

## 4. Conclusions

p16 immunostaining is used to facilitate the diagnosis of HPV-related oropharyngeal cancer. However, it is important to be aware that p16 protein overexpression may also be caused by factors other than an HPV infection. Malignant melanoma is known to express the p16 protein. Morphologically differentiating between AMM and poorly differentiated squamous cell carcinoma based on HE staining is difficult. Therefore, in cases that are pathologically diagnosed as p16-positive poorly differentiated oropharyngeal squamous cell carcinoma, it is important to rule out AMM.

## Data Availability

No datasets were generated or analysed during the current study.
